# On the Diagnosis of Diseases of the Brain, Spinal Cord, and Nerves

**Published:** 1887-12

**Authors:** 


*On the Diagnosis of Diseases of the Brain, Spinal Cord, and Nerves.* By C. W. SUCKLING, M.D. (Lond.), M.R.C.P., &c., &c. With Illustrations. London: H. K. LEWIS. 1887.

The desire of practitioners to keep themselves abreast with the continued progress of medical research has led to the delivery in many educational centres of post-graduate courses of lectures. Such a course Dr. Suckling delivered at Queen's College, Birmingham, a year ago, on the diagnosis of diseases of the nervous system, and by now publishing them he is enabled to render his knowledge and doctrine accessible to the profession at large, conferring thereby benefit upon the common weal.

The volume is addressed to busy practitioners and advanced students, and lays claim to be "a small and plainly-written work;" that is to say, that it is a grammar of diagnosis only, treatment and pathology not being included within the scope of the work. A careful perusal of these lectures has convinced us that Dr. Suckling has compiled a text-book which will be of very great value to the classes to whom it appeals, and will fill a vacuum which has hitherto remained unfilled. But we are inclined to think that, however useful in the course of the lectures themselves he may have found it to introduce accounts of illustrative cases, it would have been better to have omitted them from the book—which aims at being a synopsis exclusively—and perhaps utilised the space thus gained with more expanded details of the symptoms of certain diseases. Errors of diagnosis, and therefore also of prognosis, are frequently being made in Locomotor Ataxy, and this disease, for example, might well have been treated somewhat more in detail.

